# Building a portfolio for not-for-profit activities rather than maximizing social return on investment ratio

**DOI:** 10.12688/f1000research.123642.1

**Published:** 2022-08-19

**Authors:** Fuminobu Mizutani

**Affiliations:** 1 Department of Business Administration, Kanto Gakuin University, Yokohama, 2368501, Japan

**Keywords:** Portfolio, NFP, SROI, HHI, Non-parametric test, Core competency, Financial Engineering, Accounting

## Abstract

**Background:** An influential piece of literature on effective altruism insists that not-for-profit organizations (NFPs) should concentrate their investments on a few activities to maximize their social return on investment (SROI) ratio. However, this creates greater risk for an NFP than building a portfolio of investments in activities. This study investigates whether it is desirable for executives and contributors of NFPs to build a portfolio rather than maximize the expected SROI ratio, and if so, how to build one. Solving these questions will help the chief financial officers (CFOs) of NFPs, who serve as their agents, fulfill their obligations to contributors, who are their principals, and will help advisors provide better services for their contributors, their clients.

**Methods:** Data were collected from a ranking of NFPs, then non-parametric tests were performed on this ranking and the Herfindahl-Hirschman Index (HHI).

**Results:** The HHI are between 2013 and 2688. The results of non-parametric tests do not deny that rank and HHI are independent of each other. Most of the NFPs’ investments in activities were in accord with their core competencies.

**Conclusions:** It was found that successful executives build portfolios. The findings of this study should be sufficiently practical in helping NFP executives and contributors decide whether to build portfolios, and if so, how.

## Introduction

Social return on investment (SROI) is a hot topic in accounting; in practice, a not-for-profit organization (NFP) can calculate its SROI ratio annually, the way a profit-oriented entity calculates return on equity (ROE).

The Wall Street Journal (Feb. 2, 2022) stated that Melinda French Gates will not concentrate her donations within the Bill & Melinda Gates Foundation, but instead spread her donations across various philanthropic endeavors. We know from Tuan (2008) that this foundation has been interested in measuring SROI and other forms of social impact.

The frequently quoted MacAskill (2015) insists that NFPs should concentrate on a few activities and suggests that Oxfam and WorldVision are involved in too wide a variety of activities. Moreover, some scholars use fictional examples in which contributors concentrate their donations within one NFP with the highest SROI ratio, which would maximize the expected SROI ratio; however, some NFPs unfortunately fail to achieve their goals, and some even become embroiled in scandal, meaning that each NFP is accompanied with risk for its executives and contributors.

Building a portfolio, an often-discussed topic in finance, may fulfill the needs of NFP executives and contributors who are risk-averse. The Markowitz model, also called the Modern Portfolio Theory (MPT), advises investors of profit-oriented entities against concentrating investments within only one profit-oriented entity, and this may provide insight on how contributors to NFPs should donate.

Many finance textbooks, including Brealey *et al.* (2020), discuss agency theory. The executives of an NFP are the agents of its contributors, who are its principals. If governance of an NFP is effective, its agents fulfill its principals’ needs. If building a portfolio were desirable for principals, agents would also build a portfolio. If decisions to concentrate are desirable for contributors, they would also be desirable for NFP executives.

This paper intends to help investment professionals in decision-making, including chief financial officers (CFOs) of NFPs and advisors for contributors to NFPs, who are considering building a portfolio. A review of the literature is provided in the next section, then the data and adopted methodology, which are non-parametric tests, are described in the section after that. This is followed by results, then a discussion of the results, and finally, conclusions.

### Literature review

Gneezy *et al.* (2014) is a widely read paper on NFP financial ratios that discusses not only how to calculate them, but also how to use them. Gargani (2017) is an influential and well-written paper that also discusses risk, written by a scholar with a professional background. This paper found that SROI ratio can be calculated using the formula below:

$$\text{SROI Ratio}=\frac{\sum_{t=0}^{T}\frac{B_{t}}{{\left(1+d\right)}^{t}}}{\sum_{t=0}^{T}\frac{C_{t}}{{\left(1+d\right)}^{t}}}$$ (1)

B means net social impact and C means costs, while T means the number of years of an NFP project and t is the particular financial year.

Gargani uses a model in which only the NFP with the highest SROI ratio is selected. Yates and Marra (2017) indicate the weak points of SROI and warn that SROI may lead to radical concentration of donations in the real world.

MacAskill (2015) is a typical and practical example of literature on effective altruism based on rationality. The basis of SROI is utilitarianism, as Maier *et al.* (2015) states, and utilitarianism is a philosophical thought based on rationality. Some supporters of effective altruism are utilitarian. Singer (2015) in particular is clearly based on utilitarianism and is also widely read. MacAskill is not written from the viewpoint of an extreme risk-lover, meaning that supporters of SROI cannot neglect this paper.

On the other hand, scholars such as Pahlevani *et al*. (2021) have considered a portfolio of donations. Luenberger (2014) is a widely read textbook about financial engineering that contains explanations of the Markowitz model, a model created by Nobel laureate Harry Markowitz that has become the standard for scholars. The Markowitz model is suited to risk-averse investors, whose optimal portfolio is one that is not too concentrated.

Lukomnik and Hawley (2021) depict the Markowitz model as a bloodless model that cannot tackle social problems, implying that the Markowitz model is harmful for stakeholders. Their emphasis on social problems themselves is agreed upon by most investors and contributors, as governments around the world aim to achieve the United Nations’ Sustainable Development Goals (SDGs) with the cooperation of profit-oriented entities. However, it is doubtful that the Markowitz model is really bloodless.

Regarding methodology, Clark *et al.* (2022) show that simple methodologies sometimes bring interesting findings on economics. Flori *et al.* (2019) is an influential paper stating that the Herfindahl-Hirschman index (HHI) can be used to quantify concentration of investments. Basu *et al.* (2009), a somewhat older study, used non-parametric tests extensively, showing that accounting researchers can adopt not only parametric tests but non-parametric tests as well.

It is difficult to foresee the results of statistical methods, because of the difference between NFPs and profit-oriented entities. Angerer *et al*. (2015) shows that higher risk tolerance among contributors relates to higher contributions. Some contributors may be even risk-lovers. In contrast, mainstream investors in profit-oriented entities are obviously risk-averse.

## Methods

Due to a small sample size that makes postulating Gaussian distribution impossible, this study uses a simple methodology which utilizes non-parametric tests, which can be used for small sample sizes and do not require Gaussian distribution.

Sample NFPs were collected from NGO Advisor’s “World 200 Best SGOs” for 2021. This is a leading ranking of NFPs. NGO Advisor is a Swiss organization that provides a wealth of information on NFPs in English and French and sometimes uses the word SGO (social good organization). This abbreviation was perhaps influenced by the word *association* in French.

While the characteristics of each NFP often differ, Mercy Corps, Oxfam, and Save the Children rank high on the list and have similar characteristics. Thus, this study sampled NFPs whose activities are similar to these three NFPs, are concerned with SROI, and whose headquarters are within the English-speaking world. If detailed financial information on an NFP was difficult to collect from its annual report, it was excluded from this study.

NFPs ranked in the World 200 Best SGOs are excellent in the aspect of social impact, and executives who use SROI in such NFPs are assumed to carefully consider how to invest donations received into their activities.

Each NFP conducts several activities, and the HHI of these activities can be calculated from financial reporting. Numbers showing each NFP’s investments into each segment were gathered. This study assumes that disclosed data on activities is similar to segment reporting by profit-oriented entities, which NFP executives use in their decision-making. The objective financial information used was as the list in the next section.

Data from before the coronavirus disease (COVID-19) pandemic was used in order for this study to be applicable to a wider range of situations than the special circumstances of the pandemic. The time lag between financial information and the world ranking is not an issue for this study. Whether an NFP conducted emergency relief for the pandemic does not affect how investments in activities were made in the past, and thus it is expected that the world ranking is based on investments before the pandemic.

This study calculates the HHI of each NFP in order to see how each NFP distributes the donations received among their activities. The percentage invested in each activity within the NFP was used for this calculation. If HHI was low, it can be assumed that building a portfolio rather than concentrating donations in one area is a preferable strategy for NFPs.

This study uses Spearman’s rank correlation coefficient, which is widely used among scholars, to calculate correlation between HHI and the rank of the NFP on the world ranking. A statistically significant correlation ( *p* < 0.05) would indicate that these high-ranking NFPs have not built an optimal portfolio of donations. This study also calculates Kendall’s rank correlation coefficient, which is not widely used among scholars, as a supplement because it is known that these two statistical methods sometimes show different results.

IBM SPSS Statistics 27, a reliable application widely used in social sciences, was used to calculate these two statistical methods.

## Results

Six NFPs met the requirements of this study, meaning that the sample size was 6. Data were collected from the below documents:

• *2019 Annual Impact Report* by Mercy Corps (The left schedule of page 14.)
• *Annual Report 2018-19* by Oxfam (The right schedule of page 47.)
• *Save the Children Annual Report* 2019 by Save the Children (The upper right graph of page 23.)
• *CARE USA 2019 Annual Report* by CARE (The graph named “How We Work” of page 31.)
• *2020 Annual Report* by ChildFund (The right financial statement of page 11.)
• *Annual Report and Accounts 2018/2019* by Voluntary Service Overseas (VSO) (The upper graph of page 38.)

The name, rank, and rounded HHI of each NFP are shown in Table 1.

**Table 1.** Statistical data.

Name	Rank	Herfindahl-Hirschman index (HHI)
Mercy Corps	6	2540
Oxfam	12	2152
Save the Children	15	2610
CARE	46	2688
ChildFund	112	2274
Voluntary Service Overseas (VSO)	147	2013

The HHI of the six NFPs ranged from 2013 to 2688. There were no NFPs that met the requirements of the study below rank 148 in this ranking.

**Figure 1.**
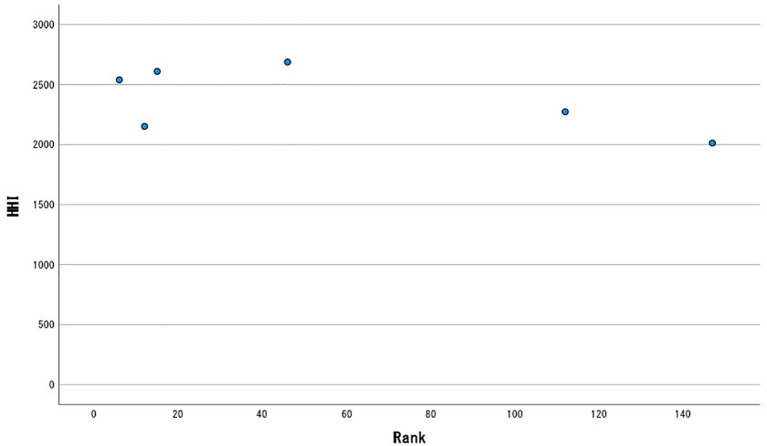
Scatter plot.

A scatter chart is shown in Figure 1. At a glance, there is no significant correlation between rank and HHI.

Spearman’s rank correlation coefficient was ρ = -0.314, statistically not significant with not only *p* ≥ 0.05, but *p* ≥ 0.1. Kendall’s rank correlation coefficient was Tau-b = -0.2, also statistically not significant at *p* ≥ 0.1. Neither of these results denies that rank and HHI are independent of each other.

Additionally, most of the six NFPs’ investments in activities were in accord with their core competencies. Even the NFP that made the largest investments in activities not in accord with its core competence among the six NFPs invested more than two-thirds in activities that were in accord with its core competence.

## Discussion

First, all six NFPs showed low HHI, showing that executives of these NFPs have built portfolios. Second, because all six NFPs had similar HHI, it would seem at a glance that an optimal portfolio exists. Statistical analysis does not deny the existence of an optimal portfolio. Finally, executives of all six NFPs appeared to prefer an optimal portfolio that consisted of activities in accord with the NFPs’ core competencies.

This suggests that skillful and rational executives build a portfolio, and that an optimal portfolio of investment into activities and an optimal portfolio of donations exist; if NFP executives neglect their core competencies, their SROI ratio will be low.

## Conclusions

These findings suggest that skillful and rational NFP executives build a portfolio of investments based on their activities. Similarly, from the statistical analysis, it can be assumed that building a portfolio of donations would benefit contributors as well. There also appears to be an optimal portfolio of donations. This study also suggests that core competencies should not be neglected, and that the Markowitz model is likely not bloodless.

This paper suggests that investment professionals should avoid overly concentrating investments, but instead build a portfolio for risk-averse clients, who make up the majority of clients. CFOs in NFPs, who serve as their agents, will be able to better fulfill their obligations to contributors, their principals, than in the past, and advisors will be able to provide better services for their clients, who are NFP contributors.

One of the limitations of this study is its small sample size. The findings of this study could be checked more rigorously if there were a method to increase the sample size. Another limitation is the widely known limits of statistical methods, which are suitable for finding differences but are not as suitable for finding similarities. The well-known statistical methods can only show that something is not denied, a slightly ambiguous indication. A method suited to finding similarities may be preferable.

Even with the limitations above, the findings of this study should be sufficiently practical in helping NFP executives and contributors decide whether to build a portfolio and, if so, how.

## Data availability

### Source data

Data was collected from a webpage of a third party “NGO Advisor”. Because NGO Advisor changed its name to thedotgood in 2022, readers can access the data from the website of thedotgood. Its URL is https://thedotgood.net/. The author utilized “World 200 Best SGOs” for 2021. In order to access the full data, registration is required. The cost for purchasing a pass varies depending on the type of the pass. The latest ranking is for 2022 in July 2022. If a reader wants to view the ranking not for 2022 but for 2021, they need to send an inquiry to thedotgood.
