# On NFPs with high social impact that avoid concentrating on a few activities

**DOI:** 10.12688/f1000research.123642.2

**Published:** 2023-10-30

**Authors:** Fuminobu Mizutani

**Affiliations:** 1 Department of Business Administration, Kanto Gakuin University, Yokohama, 2310016, Japan

**Keywords:** Portfolio, NFP, SROI, HHI, Non-parametric test, Core competency, Financial Engineering, Accounting

## Abstract

**Background:** An influential piece of literature on effective altruism insists that not-for-profit organizations (NFPs) should concentrate their investments on a few activities to maximize their social return on investment (SROI) ratio. However, this creates greater risk for an NFP than building a portfolio of investments in activities. This study investigates whether it is desirable for executives and contributors of NFPs to build a portfolio rather than maximize the expected SROI ratio, and if so, how to build one. Solving these questions will help the chief financial officers (CFOs) of NFPs, who serve as their agents, fulfill their obligations to contributors, who are their principals, and will help advisors provide better services for their contributors, their clients.

**Methods:** Data were collected from a ranking of NFPs, then non-parametric tests were performed on this ranking and the Herfindahl-Hirschman Index (HHI).

**Results:** The HHI are between 2013 and 2688. The results of non-parametric tests do not deny that rank and HHI are independent of each other. Most of the NFPs’ investments in activities were in accord with their core competencies.

**Conclusions:** It was found that successful executives build portfolios. The findings of this study should be sufficiently practical in helping NFP executives and contributors decide whether to build portfolios, and if so, how.

## Introduction

NFPs generally attempt to generate high social impact. Social return on investment (SROI) is a particularly hot topic in accounting; in practice, a not-for-profit organization (NFP) can calculate its SROI ratio annually, the way a profit-oriented entity calculates return on equity (ROE). Adoption of SROI shows the NFP pays attention to the utilitarian aspect of this. Although utilitarianism usually considers all stakeholders utility, this study’s ultimate aim is to find a way to maximize contributors’ utility through maximizing utility of their agents, namely executives of NFPs.

The Wall Street Journal (Feb. 2, 2022) stated that Melinda French Gates will not concentrate her donations within the Bill & Melinda Gates Foundation, but instead spread her donations across various philanthropic endeavors. We know from Tuan (2008) that this foundation has been interested in measuring SROI and other forms of social impact.

The frequently quoted MacAskill (2015) insists that NFPs should concentrate on a few activities and suggests that Oxfam and WorldVision are involved in too wide a variety of activities. Moreover, some scholars use fictional examples in which contributors concentrate their donations within one NFP with the highest SROI ratio, which would maximize the expected SROI ratio. Certainly, such usage of SROI could be effective based on the premise that resources are scare. However, executives of a NFP also want to maximize social impact even when they engage in activities without SROI calculations. However, some NFPs unfortunately fail to achieve their goals, and some even become embroiled in scandal, meaning that each NFP is accompanied with risk for its executives and contributors.

Building a portfolio, an often-discussed topic in finance, may fulfill the needs of NFP executives and contributors who are risk-averse. The Markowitz model, also called the Modern Portfolio Theory (MPT), advises investors of profit-oriented entities against concentrating investments within only one profit-oriented entity, and this may provide insight on how contributors to NFPs should donate.

Many finance textbooks, including Brealey *et al.* (2020), discuss agency theory. The executives of an NFP are the agents of its contributors, who are its principals. If governance of an NFP is effective, its agents fulfill its principals’ needs. If building a portfolio were desirable for principals, agents would also build a portfolio. If decisions to concentrate are desirable for contributors, they would also be desirable for NFP executives.

This paper intends to help investment professionals in decision-making, including chief financial officers (CFOs) of NFPs and advisors for contributors to NFPs, who are considering building a portfolio. A review of the literature is provided in the next section, then the data and adopted methodology, which are non-parametric tests, are described in the section after that. This is followed by results, then a discussion of the results, and finally, conclusions.

### Literature review

Various measurements to evaluate social impact have been developed in recent years. Nicholls *et al.* (2012) is a SROI guide and influences the accounting practices of British charities. This guide explains not only the financial accounting aspect of SROI but the management accounting aspect. Although some literature, ex. Bellucci *et al.* (2019) which is extensive, tackle the management accounting aspect and their findings are insightful for accounting practices, this study focuses on the financial aspect.

Gneezy *et al.* (2014) is a widely read paper on NFP financial ratios that discusses not only how to calculate them, but also how to use them. Gargani (2017) is an influential and well-written paper that also discusses risk, written by a scholar with a professional background. This paper found that SROI ratio can be calculated using the formula below:

$$\text{SROI Ratio}=\frac{\sum_{t=0}^{T}\frac{B_{t}}{{\left(1+d\right)}^{t}}}{\sum_{t=0}^{T}\frac{C_{t}}{{\left(1+d\right)}^{t}}}$$ (1)

B means net social impact and C means costs, while T means the number of years of an NFP project and t is the particular financial year.

Gargani uses a model in which only the NFP with the highest SROI ratio is selected. Yates and Marra (2017) indicate the weak points of SROI and warn that SROI may lead to radical concentration of donations in the real world. Speaking not of social impact in general but of SROI itself, whether the concentration is positive or negative for contributors is controversial and beyond the scope of this study.

MacAskill (2015) is a typical and practical example of literature on effective altruism based on rationality. The basis of SROI is utilitarianism, as Maier *et al.* (2015) states, and utilitarianism is a philosophical thought based on rationality. Some supporters of effective altruism are utilitarian. Singer (2015) in particular is clearly based on utilitarianism and is also widely read. MacAskill is not written from the viewpoint of an extreme risk-lover, meaning that executives of a NFP who want to generate high social impact cannot neglect this book.

On the other hand, scholars such as Pahlevani *et al*. (2021) have considered a portfolio of donations. Luenberger (2014) is a widely read textbook about financial engineering that contains explanations of the Markowitz model, a model created by Nobel laureate Harry Markowitz that has become the standard for scholars. The Markowitz model is suited to risk-averse investors, whose optimal portfolio is one that is not too concentrated.

Lukomnik and Hawley (2021) depict the Markowitz model as a bloodless model that cannot tackle social problems, implying that the Markowitz model is harmful for stakeholders. Their emphasis on social problems themselves is agreed upon by most investors and contributors, as governments around the world aim to achieve the United Nations’ Sustainable Development Goals (SDGs) with the cooperation of profit-oriented entities.

Taking into account the history of the Markowitz model as described in Townsend (2020), it is doubtful that this model is really bloodless. Certainly, in the past it was commonly thought that a portfolio should be only based on the pecuniary aspects of the Markowitz model. However, Nobel laureate Milton Friedman indicated the necessity of social responsibility and Markowitz himself had the same opinion as Friedman.

Through empirical research, Davis (2020) discovered that Canadian grassroots international NGOs concentrate on a few activities in the aspect of their foci and countries. Popular foci are education, health, and social services. Popular countries are India, Kenya, Haiti, and Uganda. Davis (2020) indicates Canadian international NGOs generally followed global trends.

Buchak (2023) explains risk and ambiguity from a utilitarianism perspective. Decision making depends on whether stakeholders maximize (simple) expected utility or risk-weighted expected utility. Ambiguity means stakeholders cannot know the sharp probabilities of previous results. Thus, some scholars of decision theory regard stakeholders as calculating α-maximin and they can make decisions. If the results of this study show executives of a NFP build portfolios, they are maximizing risk-weighted expected utility. Hence, if there is a pattern in portfolios, executives of a NFP are not taking random measures against ambiguity but calculated measures and the calculated measures may be α-maximin.

Regarding methodology, Clark *et al.* (2022) show that simple methodologies sometimes bring interesting findings on economics. Flori *et al.* (2019) is an influential paper stating that the Herfindahl-Hirschman index (HHI) can be used to quantify concentration of investments. As Chikoto *et al.* (2016) say, HHI is widely used in research on the revenue concentration of NFPs. Rimes *et al.* (2019) adopts HHI to examine the concentration of giving which are expenditures for contributors. HHI is appropriate for both revenue and expenditure. Basu *et al.* (2009), a somewhat older study, used non-parametric tests extensively, showing that accounting researchers can adopt not only parametric tests but non-parametric tests as well. Marklein *et al.* (2019) is a current study in the social sciences, adopting the Mann-Whitney U-Test which is a non-parametric test.

It is difficult to foresee the results of statistical methods, because of the difference between NFPs and profit-oriented entities. Angerer *et al*. (2015) shows that higher risk tolerance among contributors relates to higher contributions. Some contributors may be even risk-lovers. In contrast, mainstream investors in profit-oriented entities are obviously risk-averse.

## Methods

Due to a small sample size that makes postulating Gaussian distribution impossible, this study uses a simple methodology which utilizes non-parametric tests, which can be used for small sample sizes and do not require Gaussian distribution. Non-parametric tests can be conducted even on a small-size sample.

The research methods consist of two parts. Part one is an analysis of the world’s top NFPs. Part two is a comparison between the world’s top NFPs and more grassroots NFPs.

Sample NFPs were collected from NGO Advisor’s “World 200 Best SGOs” for 2021. This is a leading ranking of NFPs. NGO Advisor is a Swiss organization that provides a wealth of information on NFPs in English and French and sometimes uses the word SGO (social good organization). This abbreviation was perhaps influenced by the word *association* in French. Although the definitions of NFP and NGO are almost identical, the definitions of NFP and SGO are completely different. All organizations referred to in the following paragraphs are both NFPs and SGOs.

While the characteristics of each NFP often differ, Mercy Corps, Oxfam, and Save the Children rank high on the list and have similar characteristics. Thus, this study sampled NFPs whose activities are similar to these three NFPs, are concerned with SROI, and whose headquarters are within the English-speaking world. If detailed financial information on an NFP was difficult to collect from its annual report, it was excluded from this study.

According to NGO Advisor’s webpage, their ranking is from scores on NFPs’ social impact, innovation, and governance. Although it is a little difficult to evaluate in terms of innovation, good governance shows that agents fulfill principals’ needs. NFPs ranked in the World 200 Best SGOs are excellent in the aspect of social impact, and executives who use SROI in such NFPs are assumed to carefully consider how to invest donations received into their activities.

Each NFP conducts several activities, and the HHI of these activities can be calculated from financial reporting. Numbers showing each NFP’s investments into each segment were gathered. This study assumes that disclosed data on activities is similar to segment reporting by profit-oriented entities, which NFP executives use in their decision-making. The objective financial information used was as the list in the next section.

Data from before the coronavirus disease (COVID-19) pandemic was used in order for this study to be applicable to a wider range of situations than the special circumstances of the pandemic. The time lag between financial information and the world ranking is not an issue for this study. Whether an NFP conducted emergency relief for the pandemic does not affect how investments in activities were made in the past, and thus it is expected that the world ranking is based on investments before the pandemic.

This study calculates the HHI of each NFP in order to see how each NFP distributes the donations received among their activities. The percentage invested in each activity within the NFP was used for this calculation. If HHI was low, it can be assumed that building a portfolio rather than concentrating donations in one area is a preferable strategy for NFPs.

This study uses Spearman’s rank correlation coefficient, which is widely used among scholars, to calculate correlation between HHI and the rank of the NFP on the world ranking. A statistically significant correlation ( *p* < 0.05) would indicate that these high-ranking NFPs have not built an optimal portfolio of investments in activities. This study also calculates Kendall’s rank correlation coefficient, which is not widely used among scholars, as a supplement because it is known that these two statistical methods sometimes show different results.

Part two sampled NFPs with similar characteristics as samples in part one from GuideStar. The NFPs are located in Washington, D.C. and are labelled as platinum. In order to proceed with the Mann-Whitney U-Test, it is technically desirable for the sample size of more grassroots NFPs to be the same as the sample size of part one. From the high-ranked NFPs in the search results on GuideStar in 2023 September, samples with their 2019 segment information were collected.

IBM SPSS Statistics 29, a reliable application widely used in social sciences, was used to calculate these two statistical methods. The HHI of each NFP is calculated by a spreadsheet.

## Results

Six NFPs met the requirements of this study, meaning that the sample size was 6. Data were collected from the below documents:

•
*2019 Annual Impact Report* by Mercy Corps (The left schedule of page 14.)
•
*Annual Report 2018-19* by Oxfam (The right schedule of page 47.)
•
*Save the Children Annual Report* 2019 by Save the Children (The upper right graph of page 23.)
•
*CARE USA 2019 Annual Report* by CARE (The graph named “How We Work” of page 31.)
•
*2020 Annual Report* by ChildFund (The right financial statement of page 11.)
•
*Annual Report and Accounts 2018/2019* by Voluntary Service Overseas (VSO) (The upper graph of page 38.)

The name, rank, and rounded HHI of each NFP are shown in Table 1.

**Table 1.** Statistical data in part one.

Name	Rank	Herfindahl-Hirschman index (HHI)
Mercy Corps	6	2540
Oxfam	12	2152
Save the Children	15	2610
CARE	46	2688
ChildFund	112	2274
Voluntary Service Overseas (VSO)	147	2013

The HHI of the six NFPs ranged from 2013 to 2688. There were no NFPs that met the requirements of the study below rank 148 in this ranking.

**Figure 1.**
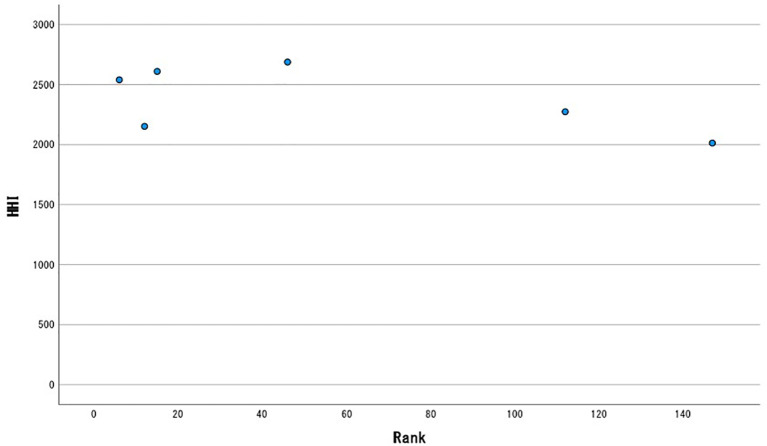
Scatter plot.

A scatter chart is shown in Figure 1. At a glance, there is no significant correlation between rank and HHI.

Spearman’s rank correlation coefficient was ρ = -0.314, statistically not significant with not only *p* ≥ 0.05, but *p* ≥ 0.1. Kendall’s rank correlation coefficient was Tau-b = -0.2, also statistically not significant at *p* ≥ 0.1. Neither of these results denies that rank and HHI are independent of each other.

Additionally, most of the six NFPs’ investments in activities were in accord with their core competencies. Even the NFP that made the largest investments in activities not in accord with its core competence among the six NFPs invested more than two-thirds in activities that were in accord with its core competence.

In part two data were collected from the following documents:

•
*2019 Annual Report* by PADF (The righthand graph on page 12.)
•
*2019 Annual Report* by Anera (The lefthand financial statement on page 10.)
•
*2019 Audited Financials* by No Kid Hungry by Share Our Strength (The statement on page 5.)
•
*2019 Audited Financial Statements* by Prosperity Now (The financial statement on page 5.)
•
*FY2019 Financial Statement* by Food & Friends (The statement on page 7.)
•
*Housing Up and Affiliates* *FY2019 Audit* by Housing Up (The statement on page 9.)

The name, and rounded HHI of each NFP are shown in Table 2. These data are designated as group B and data from the part one are designated as group A.

**Table 2.** Statistical data on part two.

Name	Herfindahl-Hirschman index (HHI)
PADF	3342
Anera	4687
No Kid Hungry by Share Our Strength	8854
Prosperity Now	3903
Food & Friends	4574
Housing Up	6207

**Figure 2.**
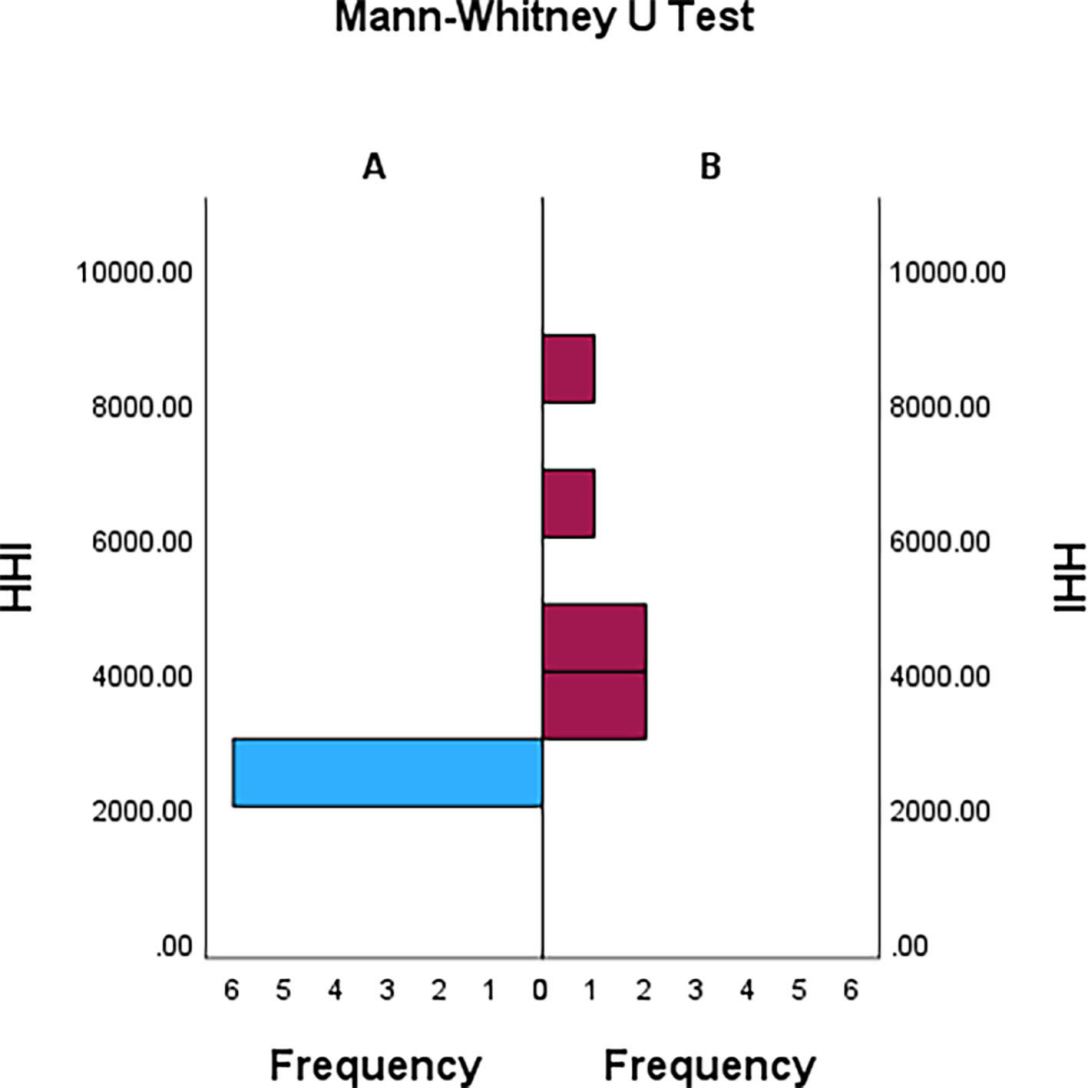
Graph about distributions of both groups.

A graph of distributions for both groups are shown in Figure 2. At a glance, the distributions of both groups are different.

U was 36 and statistically significant with p < 0.01. Thus, distributions of both groups are different.

## Discussion

Part one provided three insights. First, all six NFPs showed low HHI, showing that executives of these NFPs have built portfolios. Second, because all six NFPs had similar HHI, it would seem at a glance that an optimal portfolio exists. Statistical analysis does not deny the existence of an optimal portfolio. Finally, executives of all six NFPs appeared to prefer an optimal portfolio with the HHI ranging from 2013 to 2688 that consisted of activities in accord with the NFPs’ core competencies.

This suggests that skillful and rational executives build a portfolio, and that an optimal portfolio of investments into activities exists; if NFP executives neglect their core competencies, their social impact will be low. The existence of an optimal portfolio of investments also shows that NFP executives are not taking random measures against ambiguity but calculated measures and the calculated measures may be α-maximin.

Part two also provided another insight. The result of Mann-Whitney U-Test and Figure 2 showed that the world’s top NFPs are building portfolios while more grassroots NFPs do not necessarily build portfolios. Building a portfolio isn’t a priori for all NFPs but optimal practices conducted by the world’s top NFPs.

This suggests that following this optimal practice makes NFPs which were used not to build portfolio socially more impactful.

## Conclusions

These findings suggest that skillful and rational NFP executives avoid concentrating on only a few activities. Such executives build a portfolio of investments based on their activities. Similarly, from the statistical analysis, it can be assumed that building a portfolio of investments would benefit contributors as well. There also appears to be an optimal portfolio of investments. Besides, building portfolio is a priori for not all NFPs but is considered the optimal practice. This study also suggests that core competencies should not be neglected, and that the Markowitz model is likely not bloodless.

This paper suggests that investment professionals should avoid overly concentrating investments, but instead build a portfolio for risk-averse clients, who make up the majority of clients. CFOs in NFPs, who serve as their agents, will be able to better fulfill their obligations to contributors, their principals, than in the past, and advisors will be able to provide better services for their clients, who are NFP contributors.

Contrary to the recommendation of MacAskill (2015), the world’s top NFPs, NFPs with high social impact, avoid concentrating on a few activities. This is surprising, considering that MacAskill (2015) is essential and thoughtful literature for scholars researching NFPs. Executives of the world’s top NFPs maximize risk-weighted utility which is a particular type of utility in Buchak (2023). The statistical analysis of part two is supportive for Davis (2020) regarding grassroots NFPs.

One of the limitations of this study is its small sample size. The findings of this study could be checked more rigorously if there were a method to increase the sample size. Another limitation is the widely known limits of statistical methods, which are suitable for finding differences but are not as suitable for finding similarities. The well-known statistical methods can only show that something is not denied, a slightly ambiguous indication. A method suited to finding similarities may be preferable.

Even with the limitations above, the findings of this study should be sufficiently practical in helping NFP executives and contributors decide whether to build a portfolio and, if so, how.

## Data availability

### Source data

Data for part one was collected from a webpage of a third party “NGO Advisor”. Because NGO Advisor changed its name to thedotgood in 2022, readers can access the data from the website of thedotgood. Its URL is https://thedotgood.net/. The author utilized “World 200 Best SGOs” for 2021. In order to access the full data, registration is required. The cost for purchasing a pass varies depending on the type of the pass. The latest ranking is for 2022 in July 2022. If a reader wants to view the ranking not for 2022 but for 2021, they need to send an inquiry to thedotgood.

Data for part two was collected from a third party webpage called “Candid’s Guidestar.” Candid provides GuideStar and readers can access the data from its website. Its URL is https://www.guidestar.org/. In order to access the database, registration is required. Some of Candid’s are free in 2023.
