# Supplementary material for: On NFPs with high social impact that avoid concentrating on a few activities
Source: F1000Res. 2023 Oct 30;11:964. Originally published 2022 Aug 19. [Version 2] doi: 10.12688/f1000research.123642.2 (PMC10831226; doi:10.12688/f1000research.123642.2)
Supplement: Peer review history

# Reviewer report: Reviewer response for version 2

**DOI:** 10.5256/f1000research.157875.r219329

**Published:** 2024-01-31

**Authors:** Marco Bellucci

**Affiliations:** 1 Department of Economics and Management, University of Florence, Florence, Italy

The author has included all my suggestions, No further comments.

Is the work clearly and accurately presented and does it cite the current literature?

Partly

If applicable, is the statistical analysis and its interpretation appropriate?

Partly

Are all the source data underlying the results available to ensure full reproducibility?

Partly

Is the study design appropriate and is the work technically sound?

Partly

Are the conclusions drawn adequately supported by the results?

No

Are sufficient details of methods and analysis provided to allow replication by others?

Partly

Reviewer Expertise:

Accounting

I confirm that I have read this submission and believe that I have an appropriate level of expertise to confirm that it is of an acceptable scientific standard.

# Reviewer report: Reviewer response for version 2

**DOI:** 10.5256/f1000research.157875.r219328

**Published:** 2023-11-08

**Authors:** Linda M. Parsons

**Affiliations:** 1 The University of Alabama, Tuscaloosa, Alabama, USA

The author did a good job responding to my questions and suggestions. While the sample size is small and the analysis is simple, I think the findings are informative to those who are concerned about the need to concentrate efforts on one or a few program areas in order to provide the greatest impact on society.

 The question is an interesting one and this study provides some information about current practices.

Is the work clearly and accurately presented and does it cite the current literature?

Yes

If applicable, is the statistical analysis and its interpretation appropriate?

Yes

Are all the source data underlying the results available to ensure full reproducibility?

Yes

Is the study design appropriate and is the work technically sound?

Partly

Are the conclusions drawn adequately supported by the results?

Partly

Are sufficient details of methods and analysis provided to allow replication by others?

Partly

Reviewer Expertise:

Nonprofit accounting research using archival reports and disclosures.

I confirm that I have read this submission and believe that I have an appropriate level of expertise to confirm that it is of an acceptable scientific standard.

# Reviewer report: Reviewer response for version 1

**DOI:** 10.5256/f1000research.135771.r178726

**Published:** 2023-08-03

**Authors:** Linda M. Parsons

**Affiliations:** 1 The University of Alabama, Tuscaloosa, Alabama, USA

The author examines whether a common claim that concentrating investments (rather than building a portfolio of activities) leads to better social return on investment (SROI). This is an interesting question and, as is often the case in the nonprofit sector, involves considering the appropriateness of using corporate (profit-maximizing) techniques in nonprofit organizations. In this example, financial theory suggests a diversified portfolio is less risky and more profitable than a concentrated one. However, given the focus on SROI, perhaps dedicating nonprofit resources and expertise to a specific activity is more effective.

 The primary question/concern is the measures employed. Rather than directly measuring social impact, the author uses the ranking from a reputable international source. The assumption is that ranking of the 200 best social good organizations is an appropriate proxy for (and highly correlated with) social impact. If this is the case, a sentence or two to acknowledge this assumption and the logic of doing so would be helpful. Are there other factors (such as organizational growth or governance) that significantly affect rankings?

 The author calculates the correlation of ranking (as a proxy for impact) with a widely-accepted measure of portfolio diversity, the Herfindahl-Hirschman Index (HHI). The results show no significant association between HHI and ranking of six large nonprofits. However, later the author states that there is little variation in the HHI of the sample organizations. The lack of variation implies that both high- and lower-ranked organizations have similar investment diversity. The conclusion is that diversifying does not appear to lead to a high or low ranking, and that results “suggest that investment professionals should avoid overly concentrating investments.” I don’t think the results allow this conclusion. You show that the organizations in your sample (which are all in the top 200 internationally) do, in fact, diversify, and that this diversity is similar for those at the top of the ranking and those further down the list. What you cannot observe is whether concentrating investments would improve rankings for those at the bottom or hurt the rankings of those at the top. A more appropriate comparison (to reach your conclusion) would be a look at organizations that are not in the top 200 to see if their investments are more or less concentrated.

 I think the author can address these questions/concerns with a little more explanation of the choice of measures.

Is the work clearly and accurately presented and does it cite the current literature?

Yes

If applicable, is the statistical analysis and its interpretation appropriate?

Yes

Are all the source data underlying the results available to ensure full reproducibility?

Yes

Is the study design appropriate and is the work technically sound?

Partly

Are the conclusions drawn adequately supported by the results?

Partly

Are sufficient details of methods and analysis provided to allow replication by others?

Partly

Reviewer Expertise:

Nonprofit accounting research using archival reports and disclosures.

I confirm that I have read this submission and believe that I have an appropriate level of expertise to confirm that it is of an acceptable scientific standard, however I have significant reservations, as outlined above.

# Response

**Published:** 2023-08-04

**Authors:** Fuminobu Mizutani

**Affiliations:** Kanto Gakuin University, Japan

Thank you very much for your valuable reviewing. I will revise this manuscript as soon as possible.

# Response

**Published:** 2023-10-20

**Authors:** Fuminobu Mizutani

**Affiliations:** Kanto Gakuin University, Japan

Thank you very much for your comments. Your comments were helpful for the revision.

 Paragraph 1

 This paper’s title was changed. This paper’s focus moved from SROI to social impact in general.

 Paragraph 2

 More explanations of the data source were added in “methods”.

 Paragraph3

 Additional data were collected and another statistical analysis was conducted.

# Reviewer report: Reviewer response for version 1

**DOI:** 10.5256/f1000research.135771.r148079

**Published:** 2022-09-01

**Authors:** Marco Bellucci

**Affiliations:** 1 Department of Economics and Management, University of Florence, Florence, Italy

Thanks for the opportunity to read and review this study that investigates "whether it is desirable for executives and contributors of NFPs to build a portfolio rather than maximize the expected SROI ratio". While the topic is relevant and interesting, I believe the present version of this study would benefit from several revisions to the literature review, research design and discussion of contributions. The subject of the paper is more than relevant nowadays, but, in my view, the study also presents an important set of shortcomings that hampers, in this present version, its potential to contribute to the literature.

 From a constructive perspective, the authors could consider the following suggestions.

1. I have several concerns about the study's premises. The calculation of an SROI ratio and the construction of a portfolio are not incompatible avenues, and they are not alternatives to each other. This is especially true if we consider the usefulness of SROI as a planning and control tool. The paper is highly focused on the calculation of the SROI ratio and the selection of projects with a higher value. Still, in this way, it oversees how an organization can use the process behind the SROI methodology to foster social impact.
2. The literature review is scarce and ignores recent contributions on this topic. Moreover, the SROI methodology, the Markowitz model, and the HHI are not properly presented nor framed in the context of NFPs.
3. It is not completely clear how the author built the sample for this study, and the author should address how the criteria behind the report on the "200 Best SGOs" could affect his results. Moreover, the paper uses many terms (NGOs, NFPs, SGOs, associations) that refer to different organizations in the third sector. Furthermore, the study is based on six organizations, and this may not be enough to provide significant findings.
4. The author sometimes refers to "an optimal portfolio of donations". For whom? For the donor organization? For the beneficiaries? For the society at large? I fear this utilitarian perspective may neglect the true significance of social impact. I think the author should better clarify the contribution of this study in terms of how it can improve the selection of projects to be financed and, thus, their social impact.
5. In this version, the author does not clearly explain how he calculated the HHI and how this financial measure of market concentration can be useful to explain the choice of an "optimal portfolio" for nonprofit donors.
6. The absence of a theoretical framework led to a very brief and underwhelming discussion of results.
7. The author writes that "These findings suggest that skillful and rational NFP executives build a portfolio of investments based on their activities. Similarly, from the statistical analysis, it can be assumed that building a portfolio of donations would benefit contributors as well". However, in the present version, I do not see how the results can support this conclusion; moreover, it is difficult to know the contribution to the literature on SROI and social impact evaluation.

In general, I believe that author would need to consider deeply revising their study, particularly concerning the theoretical and managerial contribution and the research premises. I do realize that I have been critical throughout this review, but I also truly hope this feedback can help the authors to reconsider their conceptual approach to this interesting research strand.

Is the work clearly and accurately presented and does it cite the current literature?

Partly

If applicable, is the statistical analysis and its interpretation appropriate?

Partly

Are all the source data underlying the results available to ensure full reproducibility?

Partly

Is the study design appropriate and is the work technically sound?

Partly

Are the conclusions drawn adequately supported by the results?

No

Are sufficient details of methods and analysis provided to allow replication by others?

Partly

Reviewer Expertise:

Accounting

I confirm that I have read this submission and believe that I have an appropriate level of expertise to state that I do not consider it to be of an acceptable scientific standard, for reasons outlined above.

# Response

**Published:** 2022-09-02

**Authors:** Fuminobu Mizutani

**Affiliations:** Kanto Gakuin University, Japan

Thank you very much for your report. I would like to revise my manuscript after receiving other reviewers' comments.

# Response

**Published:** 2023-10-20

**Authors:** Fuminobu Mizutani

**Affiliations:** Kanto Gakuin University, Japan

Thank you very much for your comments. Your comments were helpful for the revision.

 1. This paper’s focus moved from SROI to social impact in general.

 2. The section of literature review was revised to explain SROI, Markowitz model, and HHI in the context of NFPs.

 3. The direct explanation of sampling is in paragraph 3 of “Methods” and the sampling of part two made the sampling criteria clearer.

 4. If the limitation is necessary, for contributors who are principles as explained in “Introduction”.

 5. HHI was calculated by a spreadsheet as written in “Methods”. Why optimal was explained through Buchak (2023).

 6. Some sentences were added to “Discussion”.

 7. Additional data were collected and another statistical analysis were conducted.
